# OnabotulinumtoxinA inhibits dysregulation of descending pain modulation following mild traumatic brain injury in mice

**DOI:** 10.1186/s10194-025-02159-0

**Published:** 2025-10-16

**Authors:** Robson C. Lillo Vizin, Caroline M. Kopruszinski, Janice N. Oyarzo, David W. Dodick, Ron S. Broide, Amy D. Brideau-Andersen, Mitchell F. Brin, Trent Anderson, Edita Navratilova, Frank Porreca

**Affiliations:** 1 Department of Pharmacology, College of Medicine, University of Arizona, Tucson, AZ 85724 USA; 2 Department of Collaborative Research, Mayo Clinic, Scottsdale, AZ USA; 3 Atria Academy for Science and Medicine, New York, NY USA; 4 AbbVie, Irvine, CA USA; 5 Department of Neurology, University of California, Irvine, CA USA

**Keywords:** OnabotulinumtoxinA, Conditioned pain modulation, Descending pain modulation, Diffuse noxious inhibitory controls, Post-traumatic headache, Traumatic brain injury

## Abstract

**Background:**

Diminished conditioned pain modulation, a measure of endogenous analgesia, has been reported in patients with persistent post-traumatic headache (PTH), suggesting that inefficient endogenous analgesic mechanisms may contribute to the pain. Injections of onabotulinumtoxinA into the specific regions of the head and neck have shown some benefits in treating post-traumatic headache. We investigated the potential effect of onabotulinumtoxinA on restoring the loss of descending control of nociception (DCN), a preclinical correlate of conditioned pain modulation in humans, induced by mild traumatic brain injury (mTBI) in male and female mice.

**Methods:**

We assessed DCN in a mouse weight drop model of mTBI by measuring the difference in responses to a test stimulus (i.e., latency to thermally evoked tail flick) in the absence and presence of a conditioning stimulus (i.e., injection of capsaicin in the forepaw). DCN was assessed on days 2, 4 and 14 after mTBI and on day 14 following a stress challenge elicited by exposure to bright lights, a time reflecting the persistent post-traumatic headache. OnabotulinumtoxinA (0.25 U) was injected over the cranial sutures either 2 h (early administration) or 13 days (delayed administration) post-injury.

**Results:**

mTBI transiently decreased DCN with resolution by day 14 post-injury. However, exposure to bright-light stress reinstated the loss of DCN. Sham procedures had no effects on DCN. Early administration of onabotulinumtoxinA prevented mTBI-induced loss of DCN during the transient acute period and the loss of DCN induced by bright-light stress in the persistent phase. Delayed onabotulinumtoxinA prevented bright-light stress-induced loss of DCN in the persistent phase. No sex differences were observed.

**Conclusions:**

Decreased DCN has been interpreted as a loss of endogenous analgesia that may result in pain chronification. It likely contributes to the persistent post-traumatic headache. Early or delayed administration of onabotulinumtoxinA was effective in inhibiting mTBI-induced dysregulation of DCN, indicating its potential in preventing the persistence of mTBI-induced post-traumatic headache, as well as reversing established persistent post-traumatic headache. Sexual dimorphism was not observed in these effects. Collectively, the data suggest that onabotulinumtoxinA may be beneficial in treating acute and persistent post-traumatic headache in male and female patients.

**Graphical Abstract:**

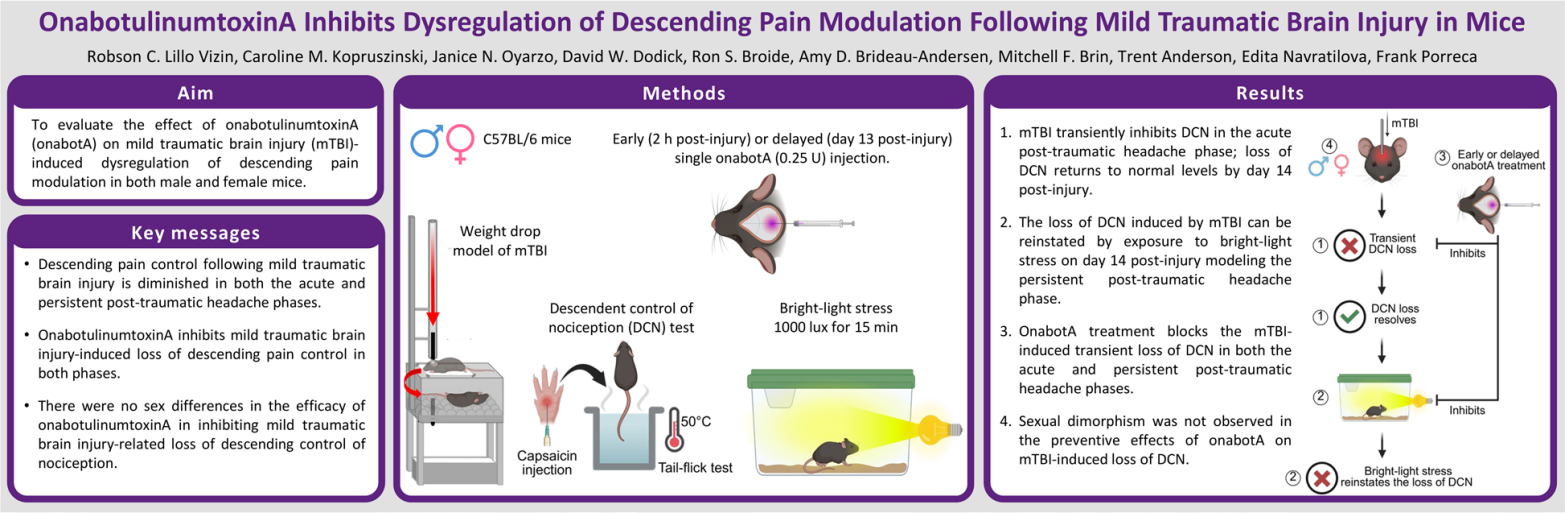

**Supplementary Information:**

The online version contains supplementary material available at 10.1186/s10194-025-02159-0.

## Background

Traumatic brain injury occurs when the brain is injured by an external force, such as a strong impact, pressure wave, or penetrating wound to the head. The estimated global incidence of TBI is 939 cases per 100,000 people, indicating that approximately 69 million people worldwide suffer a traumatic brain injury each year [1]. Among them, estimated 81%, accounting for approximately 56 million people globally, are considered mild and commonly referred to as concussion [1]. 

A common consequence of mild traumatic brain injury (mTBI) is post-traumatic headache (PTH) that occurs at a higher incidence than for more severe traumatic brain injuries [2–5]. Post-traumatic headache is a debilitating secondary headache disorder that is difficult to treat [3, 6, 7]. While most individuals recover from acute PTH within three months of onset, some continue to experience headache beyond this period and are subsequently diagnosed with persistent post-traumatic headache. Persistent PTH can present as either continuous, unremitting headache or as intermittent attacks that may be associated with provoking events including stress [3, 6, 7]. The underlying molecular and circuit mechanisms associated with mTBI-related persistent post-traumatic headache remain poorly understood but may include neurometabolic changes, activation of trigeminal sensory afferents, activation of extracranial dural afferents and central sensitization that may include disruption of descending pain modulation [6, 7]. 

Nociceptive inputs are subject to central modulation through descending pathways with cortical influence. This modulation can either inhibit or facilitate pain, depending on the context, ensuring that appropriate behavioral decisions are made to promote survival [8]. Pain modulation is thought to reflect net outcomes of engagement of central descending pain inhibitory and facilitatory circuits [8–11]. Abnormalities in brain structure and connectivity in areas relevant to descending pain modulation pathways, including the periaqueductal gray area (PAG) and the rostral ventromedial medulla (RVM), have been reported in individuals with mTBI [12–14]. The efficiency of descending pain modulation is evaluated in humans using the conditioned pain modulation test and in awake animals through measures of descending control of nociception (DCN) [6, 8, 15, 16]. The conditioned pain modulation/DCN is a pain-inhibits-pain mechanism demonstrated when the response to a test stimulus at one body site is measured in the presence of a concurrent noxious conditioning stimulus delivered to a distant body site [8–11]. In healthy individuals with efficient conditioned pain modulation, a noxious conditioning stimulus can decrease pain to the test stimulus (i.e., elicit endogenous analgesia). Importantly, reduced conditioned pain modulation has been associated with many persistent pain conditions, suggesting that a decreased response can enhance and sustain pain and may increase vulnerability to develop chronic or persistent pain [8–11]. Loss or diminished conditioned pain modulation has also been reported in patients with persistent PTH [15, 17, 18]. Therefore, inefficient endogenous analgesic mechanisms may exacerbate PTH. Decreased DCN has been observed in rodent models of traumatic brain injury [19–21], including the mouse mTBI model previously reported by our laboratory and employed in the present studies [22]. 

There is currently no approved treatment specifically for post-traumatic headache, and management strategies usually rely on medications used for tension-type headaches or migraines [6]. OnabotulinumtoxinA (BOTOX^®^), typically administered in a series of 31 injections in specific areas of the head and neck, is approved for the prevention of chronic migraine [23, 24]. We recently reported that in male mice subjected to mTBI, supracranial administration of onabotulinumtoxinA early after injury blocked mTBI-induced transient cutaneous cephalic allodynia, a measure of acute PTH. This treatment also prevented the subsequent vulnerability to stress-induced reinstatement of cephalic allodynia, a behavioral measure reflecting persistent PTH [25]. In addition, we showed that delayed onabotulinumtoxinA administration, after the resolution of transient mTBI-induced allodynia, inhibited allodynia elicited by stress during the persistent PTH [25]. However, the effects of onabotulinumtoxinA on central mechanisms promoting endogenous analgesia during the acute and persistent phases of PTH remain unknown. Given that impaired conditioned pain modulation/DCN can reduce endogenous analgesia and increase the risk for chronic or persistent pain [8–11], pharmacological strategies that restore descending pain modulation are desirable.

In the present study, we hypothesized that supracranial onabotulinumtoxinA would inhibit the loss of endogenous pain modulation induced by mTBI. To test this hypothesis, we evaluated the efficacy of a single early or delayed supracranial administration of onabotulinumtoxinA in preventing the loss of DCN during both the acute and persistent PTH phases following mTBI. Additionally, we explored the possibility of sexual dimorphism in mTBI-induced loss of DCN and/or in the efficacy of onabotulinumtoxinA.

## Methods

### Animals

A total of 153 female and 221 male C57BL6/J mice, 9- to 12-weeks old, from the Jackson Laboratory (Sacramento, CA, USA) were used in this study. Housing conditions consisted of a 12-h light/dark cycle (lights on at 7 a.m.) in a climate- and humidity-controlled environment with food and water provided *ad libitum*. This manuscript was prepared in accordance with the ARRIVE guidelines. Every effort was made to minimize the number of animals and their suffering. Animals were randomly assigned to their conditions/treatments. Investigators were blinded to treatments and experimental groups.

### Mild traumatic brain injury procedure

Mild traumatic brain injury was performed as reported previously by our group [22, 25–27]. Mice were lightly anaesthetized and laid with their ventral surface and in a prone position on an elevated sheet of tissue paper situated over a plexiglass apparatus with soft sponge at the bottom. A metal guide tube was directed to the top of the mouse skull between the ears to ensure standardized placement of the weight drop. A 100 g weight was released from a height of 94 cm onto the closed and unfixed skull, resulting in a concussive impact to the head, pushing the mouse through the tissue paper and flipping it down to land on the soft sponge. All mTBI mice experienced both rotational and linear head forces, mimicking to some degree common concussion injuries that involve free head rotation in humans [28, 29]. Sham animals were anaesthetized and placed on the tissue paper stage but did not receive the concussive impact or rotational flip. Following the procedure, the righting reflex was recorded, and mice were placed back in their home cages and allowed to recover. Only mice that showed righting reflex within 5 min of the procedure onset (i.e., > 90%) were included in the study [22, 25, 26]. 

### Drugs

Capsaicin (Tocris, Bristol, UK) was dissolved in 10% Tween 80, 10% ethanol and 80% saline to a 0.25% (0.25 µg/µL) concentration just before the injection and kept on ice. Animals received subcutaneous injection of 10 µL of capsaicin into the right forepaw under light isoflurane anesthesia [22]. OnabotulinumtoxinA (Abbvie, North Chicago, IL, USA) was reconstituted with 10 mL of sterile, preservative-free saline to obtain a stock solution with a concentration of 10 U/mL. This stock solution was refrigerated and used within 24 h. Prior to administration, the stock solution was further diluted with saline to achieve a working concentration of 5 U/mL. Under light isoflurane anesthesia, a single injection of onabotulinumtoxinA working solution (0.25 U in 50 µL saline) was administered subcutaneously into the supracranial region distributed over the sagittal/lambdoid sutures. This protocol ensures no systemic effects [25] and was optimized as a simplified version of the localized treatment used clinically for migraines. Control animals received 50 µL of saline vehicle. OnabotulinumtoxinA and vehicle were injected either 2 h (early) or 13 days (delayed) post mTBI or sham procedure.

### Bright-light stress

Bright-light stress was performed as previously described [25–27] to produce a stress without significant cutaneous allodynia in sham mice. Unrestrained mice were exposed for 15 min to bright-light stress induced by two 2000 lumens LED work lights placed on both sides of their home Plexiglass cages (light intensity in the cage area was approximately 1000 lx).

### Descending control of nociception

DCN was performed as previously described [22, 30]. Animals were gently held by the experimenter, and half of the distal tail was immersed in a 50 °C hot-water bath (Thermo Fisher Scientific, Waltham, MA, USA). The tail-flick response was used as the test stimulus. The time required for the animals to flick their tails established the baseline latency. A cut-off of 15 s was established to avoid tissue damage. Capsaicin (0.25%/10 µL) was injected into the right forepaw and serves the conditioning stimulus. The tail-flick latency was then measured at 20, 40, 60, 90, 120 and 180 min post-capsaicin injection. Percent DCN response was calculated as (TL – BL) ¸ (cutoff – BL) ×100%; where TL is the tail-flick latency at 40 min after capsaicin injection (the peak of tail-flick antinociception); BL is the baseline tail-flick latency, and cutoff is 15 s.

### Experimental design

Male and female mice received either a mTBI or a sham procedure, followed 2 h later by supracranial administration of onabotulinumtoxinA or saline. DCN was evaluated in separate cohorts of mice on day 2 (Experiment 1) and on day 4 (Experiment 2) post-injury. Physiological restoration of DCN was assessed on day 14 post-injury without exposure to bright-light stress (Experiment 3). DCN was also evaluated on day 14 immediately after exposure to bright-light stress in separate cohorts of mice treated with onabotulinumtoxinA or saline either 2 h (Experiment 4) or 13 days (Experiment 5) after mTBI.

### Statistical analysis

Time-course experiments for tail-flick latency were analyzed by two-way repeated-measures ANOVA, followed by Tukey’s multiple comparisons test. DCN responses at 40 min were analyzed by one-way ANOVA, followed by Tukey’s multiple comparisons test. Two-group comparisons were analyzed using an unpaired two-tailed Student’s t-test. Statistical analyses were performed using GraphPad Prism 10 (GraphPad Software, La Jolla, CA). Data are presented as mean ± SEM and statistical significance was set at *P* < 0.05. Statistical details are summarized in the Supplementary Table 1.

## Results

### Supracranial administration of onabotulinumtoxinA early after mTBI prevents injury-induced loss of descending control of nociception

**Fig. 1:**
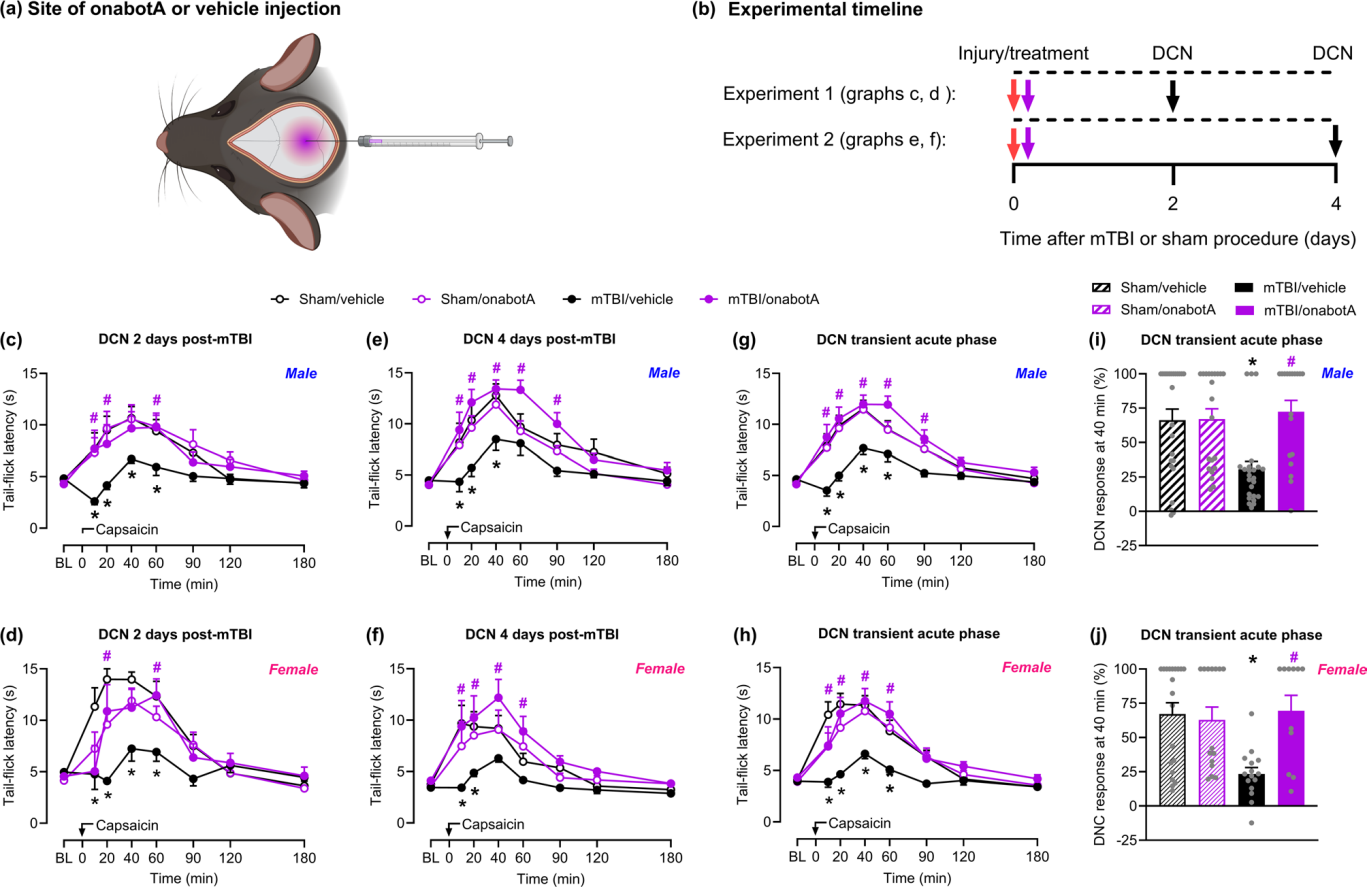
OnabotulinumtoxinA (onabotA) prevents mTBI-induced transient loss of DCN. **a** Illustration of onabotA injection site over the sagittal and lambdoid suture intersection on the mouse skull. (**b**) Timeline of testing for Experiments 1 and 2. Mice were subjected to mTBI or sham procedure and, 2 h later, treated with a single supracranial injection of onabotA (0.25U/50 µL) or vehicle (saline, 50 µL). Baseline (BL) tail-flick latencies of male and female mice, respectively, were assessed on (**c**,** d**) day 2 and (**e**,** f**) day 4 post-mTBI or sham procedure, followed by subcutaneous injection of capsaicin (0.25%/10 µL) into the right forepaw. Tail-flick evaluation was performed over a 3-h (180 min) period post-capsaicin injection. Different cohorts of mice were used for each day of evaluation. Compilation of data from both day 2 and day 4 post-procedure for (**g**) male and (**h**) female mice. Percentage of DCN response 40 min after forepaw capsaicin injection, compiled from both day 2 and day 4 post-procedure for (**i**) male and (**j**) female mice. Data are presented as mean ± SEM and analyzed using two-way repeated-measures ANOVA or one-way ANOVA followed by Tukey’s multiple comparison test, with *P < 0.05 mTBI/vehicle vs. sham/vehicle and #*P* < 0.05 mTBI/onabotA vs. mTBI/vehicle (*n* = 5–24). Details of statistical analyses are found in Supplementary Table 1

We evaluated the effects of onabotulinumtoxinA treatment administered 2 h after mTBI/sham injury into the supracranial region (Fig. 1a) on DCN. We selected to test these effects at 2 and 4 days post-mTBI (Fig. 1b) that correspond to our previously reported times of peak cephalic allodynia we described as a transient acute PTH phase [25, 26]. Baseline tail-flick latencies were indistinguishable between the groups, confirming that neither mTBI nor onabotulinumtoxinA had any effect of tail withdrawal responses. Capsaicin administration increased tail-flick latency in sham-treated males (Fig. 1c, e) and females (Fig. 1d, f), demonstrating effective DCN. In these sham groups, no difference in capsaicin-induced augmentation of tail-flick latency was observed between saline and onabotulinumtoxinA treatments. Compared to the sham animals, saline treated mTBI mice showed significantly reduced augmentation of tail-flick latency after capsaicin injection on both days 2 (Fig. 1c, d) and 4 (Fig. 1e, f) post-injury, indicative of a significant loss of DCN. Conversely, in mTBI mice treated with onabotulinumtoxinA, capsaicin induced a pronounced DCN response on both days (Fig. 1c-f), as indicated by tail-flick latencies similar to the respective sham groups. Because DCN responses assessed on days 2 and 4 were comparable, we compiled the data from both days to provide an average measure of endogenous analgesic efficiency during the transient acute pain phase with increased statistical power to allow comparisons between sexes (Fig. 1g, h). Our data reveal no sex differences in mTBI-induced loss of DCN compared to shams, which was completely prevented in both male (Fig. 1g) and female (Fig. 1h) mice by onabotulinumtoxinA. The prevention of mTBI-induced DCN loss by early onabotulinumtoxinA treatment was particularly evident 40 min after capsaicin injection, during peak tail-flick antinociception, in both males (Fig. 1i) and females (Fig. 1j).

### Mild traumatic brain injury-induced loss of DCN is transient and DCN returns to baseline by day 14 post-injury

**Fig. 2:**
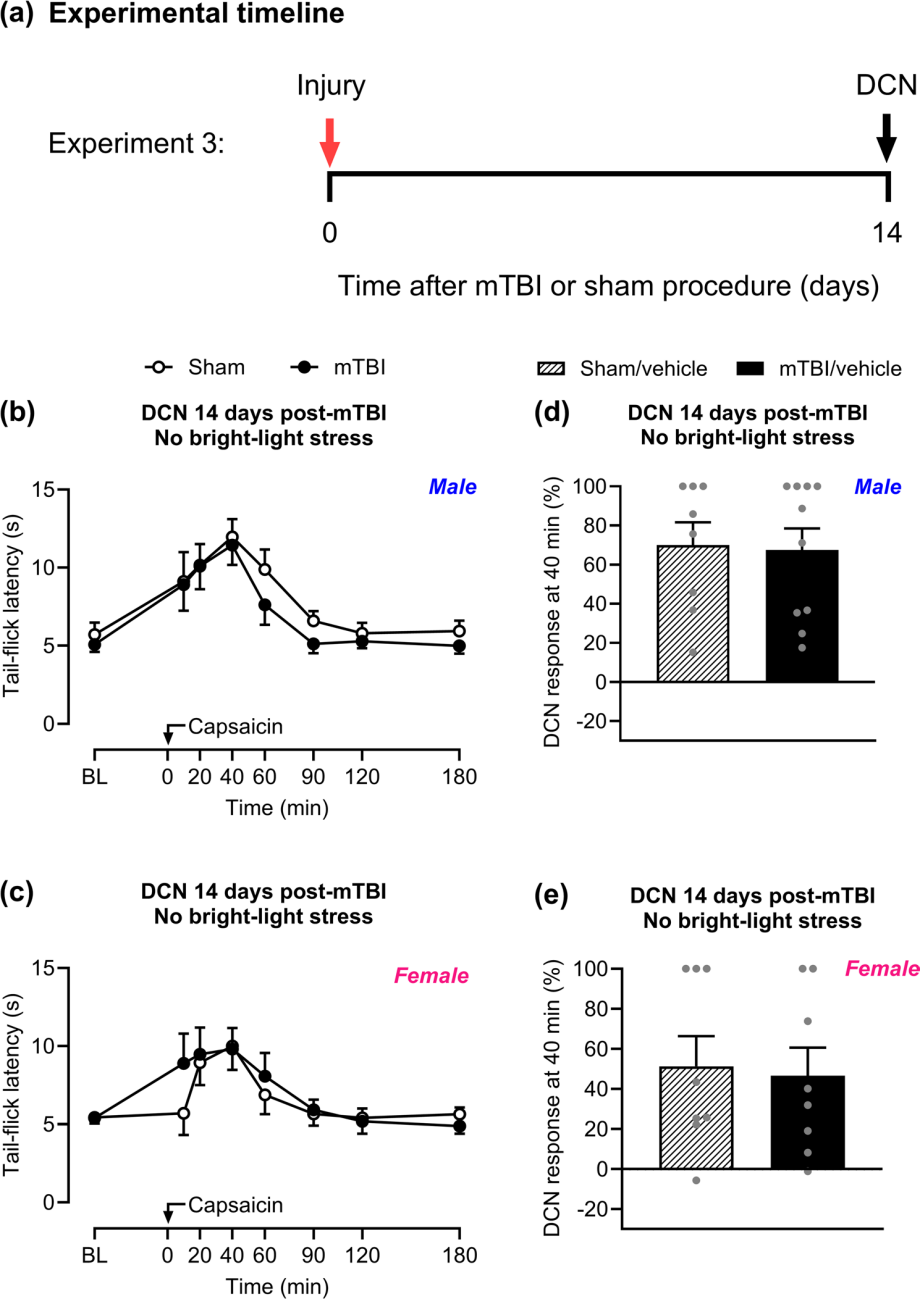
No loss of DCN is observed on day 14 following mTBI in the absence of a stressor. **a** Timeline of testing for Experiment 3. Mice were subjected to mTBI or sham procedure. On day 14 post-injury, baseline (BL) tail-flick latency was assessed in the absence of a stressor (bright-light stress). Capsaicin (0.25%/10 µL) was then injected into the right forepaw of (**b**) male and (**c**) female mice, and tail-flick responses were evaluated over a 180 min period. Percentage of DCN response 40 min after forepaw capsaicin injection in (**d**) male and (**e**) female mice. Data are presented as mean ± SEM and analyzed using two-way repeated-measures ANOVA followed by Tukey’s multiple comparison test or unpaired two-tailed Student’s t-test (*n* = 8–10). Details of statistical analyses are in Supplementary Table 1

We investigated if the loss of DCN persists through day 14 after mTBI (Fig. 2a). Following capsaicin injection, no difference in tail-flick latency was observed between sham and mTBI mice in either male (Fig. 2b) or female (Fig. 2c) groups, demonstrating effective DCN at this timepoint. The peak of the analgesic effect was observed 40 min after capsaicin injection (Fig. 2d, e).

### Bright-light stress reinstates mTBI-induced loss of descending control of nociception

**Fig. 3:**
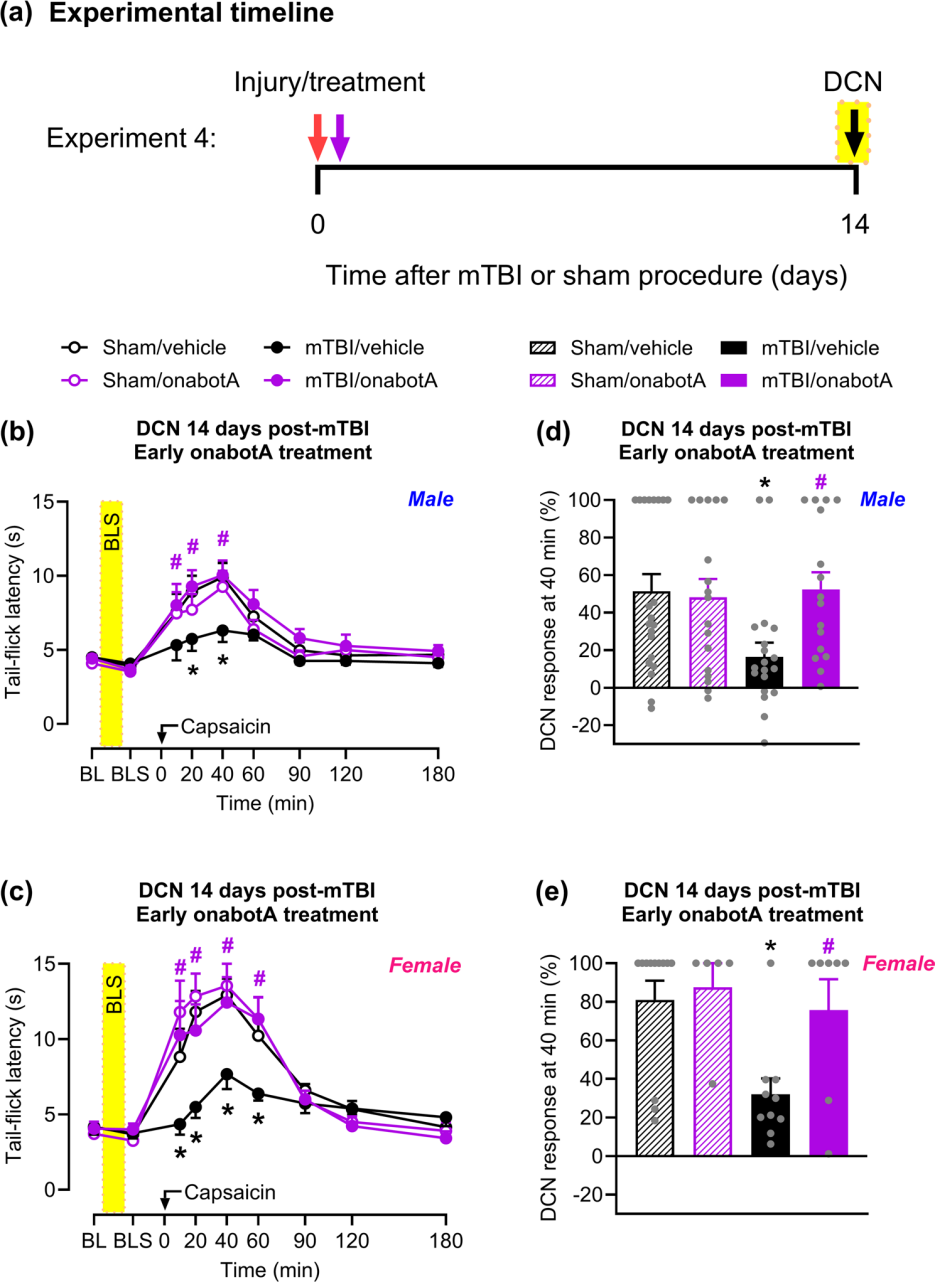
Administration of onabotA early after mTBI prevents bright-light stress-induced loss of DCN. **a** Timeline of testing for Experiment 4. Mice were subjected to mTBI or sham procedure; 2 h later they were treated with a single supracranial injection of either onabotA (0.25U/50 µL) or vehicle (saline, 50 µL). On day 14 after the procedure, tail-flick latency was assessed before (baseline, BL) and after bright-light stress exposure for 15 min (indicated by yellow-shaded areas). Capsaicin (0.25%/10 µL) was then injected into the right forepaw of (**b**) male and (**c**) female mice, and tail-flick latency was evaluated over a 180 min period. Percentage of DCN response 40 min after forepaw capsaicin injection in (**d**) male and (**e**) female mice. Data are presented as mean ± SEM and analyzed using two-way repeated-measures ANOVA or one-way ANOVA followed by Tukey’s multiple comparison test, with *P < 0.05 mTBI/vehicle vs. sham/vehicle and #*P* < 0.05 mTBI/onabotA vs. mTBI/vehicle (*n* = 5–21). See Supplementary Table 1 for details of statistical analyses

In our previous studies, we reported the resolution of cutaneous cephalic allodynia by day 13 post-mTBI [25, 26] and that allodynia returned following exposure to stressors, including bright light, suggestive of persistent PTH [25, 26]. Here, we tested if the loss of DCN in mice previously subjected to mTBI can also be reinstated by a 15 min exposure to bright-light stress (Fig. 3a). Following bright-light stress, saline-treated mTBI male (Fig. 3b) and female (Fig. 3c) mice demonstrated attenuated DCN response compared to the sham groups. Loss of DCN response induced by mTBI peaked at 40 min post-capsaicin injection (Fig. 3d and e).

### Early onabotulinumtoxinA treatment of mTBI mice prevents loss of descending control of nociception following bright-light stress

A single onabotulinumtoxinA injection given 2 h post-injury prevented bright-light stress-induced loss of DCN in mTBI mice on day 14 post-injury. This was evidenced by increased tail-flick latency post-capsaicin injection comparable to that of sham controls in both males (Fig. 3b) and females (Fig. 3c). The peak of the DCN effect was at 40 min post-capsaicin injection (Fig. 3d, e). No meaningful difference in capsaicin-induced increase in tail-flick latency was observed between saline and onabotulinumtoxinA treatments in either male (Fig. 3b, d) or female (Fig. 3c, e) sham mice.

### Delayed onabotulinumtoxinA treatment inhibits bright-light stress-induced loss of descending control of nociception

**Fig. 4:**
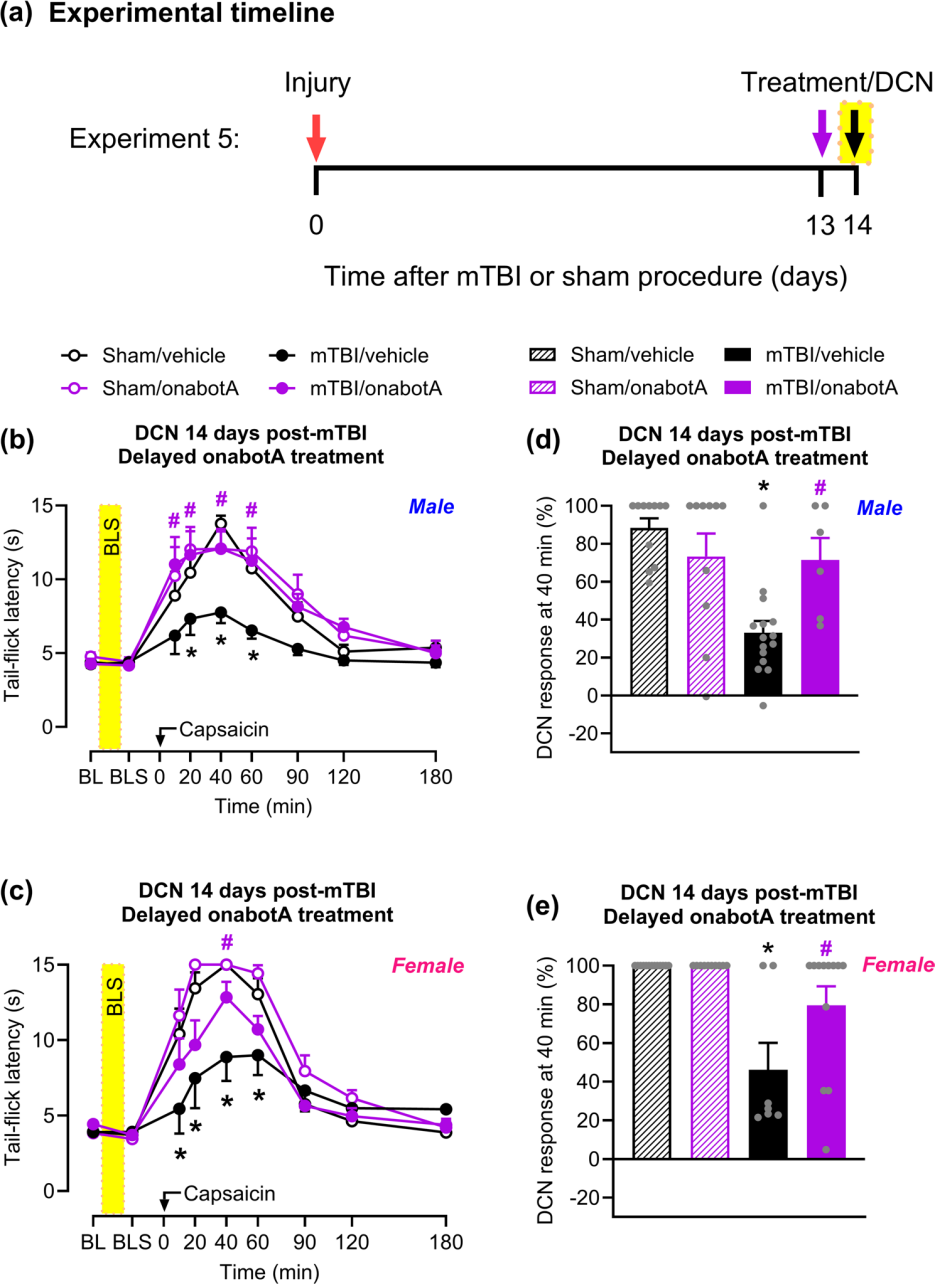
Delayed onabotA treatment prevents bright-light stress-induced loss of DCN. **a** Timeline of testing for Experiment 5. Mice were treated with a single supracranial injection of either onabotA (0.25U/50 µL) or vehicle (saline, 50 µL) on day 13 after mTBI or sham procedure. 24 hours later, on day 14, tail-flick latency was assessed before (baseline, BL) and after bright-light stress (indicated by yellow-shaded areas). Capsaicin (0.25%/10 µL) was then injected into the right forepaw of (**b**) male and (**c**) female mice, and tail-flicks were continuously evaluated over a 180 min period. Percentage of DCN response 40 min after capsaicin in (**d**) male and (**e**) female mice. Data are presented as mean ± SEM and analyzed using two-way repeated-measures ANOVA or one-way ANOVA followed by Tukey’s multiple comparison test, with *P < 0.05 mTBI/vehicle vs sham/vehicle and #*P* < 0.05 mTBI/onabotA vs mTBI/vehicle (*n* = 6–15). Details of statistical analysis are in Supplementary Table 1.

We next evaluated whether delayed administration of onabotulinumtoxinA, on day 13 post-injury, 24 h before bright-light stress and behavioral evaluation, would prevent stress-induced loss of DCN (Fig. 4a). Corroborating our findings (Fig. 3), on day 14 post-mTBI, bright-light stress reinstated the loss of DCN in both male (Fig. 4b) and female (Fig. 4c) mice treated with saline. This was indicated by a reduced tail-flick latency following capsaicin injection compared to sham, with the peak effect at 40 min post-capsaicin injection (Fig. 4 d, e). Delayed onabotulinumtoxinA injection prevented bright-light stress-induced loss of DCN in both male (Fig. 4b) and female (Fig. 4c) mTBI mice, with the peak effect at 40 min post-capsaicin injection (Fig. 4 d, e). No difference in capsaicin-induced tail-flick latency was observed between saline and onabotulinumtoxinA treatments in sham mice (Fig. 4b-e).

## Discussion

Central circuits can facilitate or inhibit nociception through descending pain modulatory pathways, respectively allowing for attending or escape behaviors depending on context. Multiple chronic pain conditions [31–34] including primary headache disorders [11, 35–38] and post-traumatic headache [15, 17, 18] are characterized by a net loss of descending pain inhibition, measured in humans as conditioned pain modulation. It has been suggested that the net loss of descending pain inhibition leading to reduced endogenous analgesia, is due to enhanced descending facilitation that may swamp normal descending inhibition [22, 30, 39–42]. Importantly, loss of endogenous analgesia may be a key mechanism promoting chronification of pain and chronic pain itself may further weaken conditioned pain modulation function [9, 11, 43]. 

The DCN originates from the brainstem reticularis dorsalis subnucleus and interacts with the PAG and the RVM [44]. Preclinical studies and neuroimaging in humans reveal engagement of circuits above the brainstem, especially the prefrontal and cingulate cortices and the amygdala, during conditioned pain modulation/DCN [45]. The involvement of higher brain centers could explain the role of emotional and cognitive factors like stress in dysfunctional CPM in chronic pain [46]. Our recent studies found that endogenous kappa opioid receptor (KOR) signaling in the anterior cingulate cortex and right central nucleus of the amygdala could drive DCN loss in animals with neuropathic pain. Dynorphin/KOR signaling is part of the stress response, suggesting that this mechanism could promote the loss of DCN and progression to chronic pain in other stress-related pain conditions.

Preserving central pathways that promote endogenous analgesia could therefore be crucial in preventing the progression of pain conditions to more chronic states such as persistent post-traumatic headache. Disrupted descending modulation following mild traumatic brain injury may represent a potential prospective indicator of pain progression and/or persistence [8, 9, 13, 15]. Indeed, a previous study has prospectively demonstrated increased likelihood of development of chronic pain from scheduled surgeries in patients with weak conditioned pain modulation [47]. 

Previously, we reported that a single supracranial administration of onabotulinumtoxinA 2 h after mTBI fully blocked both mTBI-induced transient cutaneous cephalic allodynia and stress-induced allodynia, interpreted as reflecting acute and persistent PTH, respectively [25]. However, the relationship between mTBI-induced cephalic allodynia and descending modulation of nociception remained uncertain. In the present study we asked whether a treatment that blocks mTBI-related cephalic allodynia could also be effective in preventing or restoring dysregulation of descending pain modulation. We found that (a) consistent with previous reports [19–22], mTBI inhibits descending control of nociception; (b) inhibition of DCN is transient and is reestablished to normal levels by day 14 post-injury, a timepoint at which transient cephalic allodynia has also resolved in this model [25]; (c) mTBI-induced loss of DCN, like cephalic allodynia, can be reinstated by bright-light stress; (d) early onabotulinumtoxinA treatment blocks mTBI-induced transient loss of DCN as early as 2-days post-treatment and prevents subsequent bright-light stress-induced loss of DCN; (e) delayed treatment with onabotulinumtoxinA, at times at which DCN is normal but the mice are vulnerable to stress, prevents subsequent bright-light stress-induced loss of DCN; and (f) we did not observe any consistent sexual dimorphism in the effects of onabotulinumtoxinA on prevention of loss of DCN following mTBI.

We have previously reported that following resolution of mTBI-induced cephalic allodynia, pain behaviors can be reinstated selectively in injured, but not sham, animals by exposure to a psychological stressor, such as bright light [25, 26]. The development of a sensitized state where cephalic allodynia can be triggered by an external stress stimulus appears relevant to persistent PTH as both stress and bright lights are known to exacerbate PTH in humans [48]. In agreement with our previous work [22] and others [19–21], we observed a loss of the DCN after mTBI in both male and female mice. However, this loss was transient and at day 14 post injury, DCN levels were restored nearly to baseline. Effective DCN was therefore observed at timepoints that coincided with the resolution of cephalic allodynia [25, 26], a symptom that is also observed in patients with PTH [49]. Importantly, the DCN is again diminished in mTBI mice after exposure to stress, consistent with our observations that stress reinstated cephalic allodynia. Impaired descending pain modulation may be a pathophysiological consequence of central sensitization that amplifies a subthreshold afferent input likely promoted by stress, resulting in pain behaviors such as PTH [6]. 

OnabotulinumtoxinA, is an FDA-approved drug for the prevention of chronic migraine [23, 24]. For migraine, onabotulinumtoxinA is injected into the muscles of head and neck, which are associated with the sensory innervation of the scalp, face, and cervical region [50]. We administered onabotulinumtoxinA over the sagittal/lambdoid sutures in mice to evaluate possible efficacy in blocking mTBI-related loss of DCN. A single administration of onabotulinumtoxinA 2 h after mTBI inhibited transient loss of DCN as early as 2-days post-treatment and additionally prevented subsequent loss of DCN induced by bright-light stress. This rapid effect of onabotulinumtoxinA was also previously observed for this model in reducing cephalic cutaneous allodynia as early as 1-day (first timepoint measured) post-dosing. ^25^ Together, these preclinical results suggest that the effect of toxin on sensory targets may be almost as rapid as its effect on preclinical motor targets [51, 52] and is consistent with the rapid effect of onabotulinumtoxinA observed clinically for chronic migraine treatment [53]. 

OnabotulinumtoxinA was effective in inhibiting dysregulation of DCN when administered early after a mTBI suggesting potential protective benefits that could be especially relevant to patients with increased vulnerability to developing persistent PTH such as individuals with underlying primary headache disorders [54]. The timing of onabotulinumtoxinA administration is an especially important variable as pharmacological treatments shortly after a mTBI may not be practical in many patients. Importantly, we also found that delayed administration of onabotulinumtoxinA prevented stress-induced loss of DCN. This observation aligns with our previous work, showing that delayed onabotulinumtoxinA treatment prevents persistent PTH-related cephalic allodynia [25] and suggests benefit in restoring net descending inhibition with immediate inhibition of persistent PTH and possible time-related wind-down to normal net inhibitory modulation of nociceptive inputs. This possibility is supported by preliminary clinical observations that delayed onabotulinumtoxinA treatment results in favorable outcomes in patients with established persistent PTH [55, 56]. 

Clinically, migraine headache is believed to result from activation of meningeal nociceptors [57–59]. mTBI may activate both extracranial and meningeal sensory fibers, conveying information to deeper brain areas through the trigeminal nerve. These possibilities are consistent with the observed preclinical preventive effects of onabotulinumtoxinA on mTBI-related cephalic allodynia [25] and loss of DCN in this model. The effect on trigeminal sensory fibers is further supported by functional evidence showing that peripheral administration of onabotulinumtoxinA (a) suppressed the responses of meningeal nociceptors to intracranial dural stimulation with TRPV1 and TRPA1 agonists [60] and (b) inhibited the activation and sensitization of central trigeminovascular neurons by cortical spreading depression [61, 62]. OnabotulinumtoxinA is believed to impair critical pain signaling processes including the ability of nerve terminals to express pronociceptive ion channels in the synaptic membrane [63] and the ability of nociceptors to release pro-inflammatory neuropeptides and neurotransmitters such as calcitonin gene-related peptide (CGRP) [64] and prostaglandin E2 (PGE2) [65]. 

Implications of CGRP in the trigeminal pain circuit on the pathophysiology of PTH have been reported preclinically [66, 67]. Consistent with this, our previous studies demonstrated that continuous systemic treatment with an anti-CGRP monoclonal antibody prevented the transient cephalic allodynia and loss of DCN after mTBI as well as subsequent bright-light stress-induced cephalic allodynia [22, 26, 68]. In the clinic, PTH often mimics primary headaches, typically manifesting as either tension-type or migraine-like headaches [6]. Phenotypic overlap between PTH and migraine suggests shared biological mechanisms [6, 12, 69, 70]. In this sense, the interictal plasma level of CGRP in the peripheral blood may predict the efficacy of onabotulinumtoxinA treatment – patients with CGRP levels above the threshold of 72 pg/ml had a 28-fold higher probability to respond to onabotulinumtoxinA treatment [71]. In another later study, serum levels of CGRP >50 ng/ml were associated with better response to onabotulinumtoxinA treatment [72]. Supporting these findings, onabotulinumtoxinA was demonstrated to reduce circulating CGRP levels in migraine patients [73, 74], especially in patients with high levels before the onabotulinumtoxinA treatment [73]. Collectively, these studies suggest that onabotulinumtoxinA may inhibit the activation of the trigeminal nociceptive pathway by blocking the release of CGRP as well as other neurotransmitters relevant to the induction of PTH and mTBI-induced loss of DCN/conditioned pain modulation.

OnabotulinumtoxinA has been suggested to preferentially inhibit the activation and sensitization of nonmyelinated peripheral nerve fibers (i.e., C-fibers) to prevent nociceptive signaling. Central sensitization correlates clinically with cutaneous allodynia and is reduced by inhibition of CGRP-positive C-fibers [75, 76]. Thus, onabotulinumtoxinA effects on peripheral C-fibers and CGRP may inhibit the initial mTBI-induced development of central sensitization in this model providing an explanation for the long-lasting effects of an early single onabotulinumtoxinA injection. These effects include not only prevention of cephalic allodynia and transient loss of DCN in the acute phase, but also stress-induced reinstatement of both of these outcomes in the persistent phase. Consistent with this interpretation, evidence suggests that onabotulinumtoxinA prevents central sensitization in migraine, clinically measurable as a reduction in extracephalic allodynia, by modulating overall nociceptive transmission and reducing pain signals between central trigeminovascular neurons and other brain regions involved in pain processing [77]. It is worth noting that while CGRP plays a key role in establishing a sensitized state that heightens susceptibility to headache triggers, the pain-like response associated with persistent PTH and loss of DCN is not solely dependent on CGRP. Previous research from our group showed that administering a monoclonal CGRP antibody after the resolution of transient cutaneous cephalic allodynia did not block stress-induced pain behaviors in mice with prior mTBI [26], implying the involvement of other CGRP-independent mechanisms in the efficacy of onabotulinumtoxinA to abort persistent post-traumatic symptomatology and ameliorate mTBI-induced impairment of DCN.

One limitation of our study is that although we did not find any major sex differences in the severity of mTBI-induced DCN loss or in the efficacy of onabotulinumtoxinA, it could be due to insufficient power to detect subtle differences. An additional limitation is that we did not design our study to determine potential differences due to the estrus cycle in females. A recent study found that recovery after TBI in female mice depends on the stage of the estrus cycle [78]. Future investigations will determine the role of the estrus cycle in the mTBI-induced loss of DCN. Our study also did not investigate the brain mechanisms that promote the loss of DCN. It is assumed that the DCN originates from the brainstem reticularis dorsalis subnucleus and interacts with the PAG and RVM [44]. Preclinical studies and neuroimaging in humans reveal engagement of circuits above the brainstem, especially the prefrontal and cingulate cortices and the amygdala, during conditioned pain modulation/DCN.^45^ The higher brain centers could explain the effect of emotional and cognitive factors like stress in dysfunctional CPM in chronic pain [46]. In this regard, our recent studies found that endogenous dynorphin/KOR signaling in the anterior cingulate cortex and right central nucleus of the amygdala could drive DCN loss in animals with neuropathic pain [39, 41]. Whether dynorphin/KOR signaling which is part of the stress response, could promote the loss of DCN and progression to chronic pain in acute and chronic PTH will be determined in future studies. Finally, it is important to acknowledge that animal models have limitations in capturing the full complexity of human PTH. It should be noted that studies in animal experiments have shorter timelines compared to human conditions possibly complicating translation of these findings to the clinic [79, 80]. 

## Conclusions

In summary, our findings suggest that both early and delayed onabotulinumtoxinA treatments mitigate mild traumatic brain injury-induced impairment in descending pain modulation. Restoration of endogenous analgesia by onabotulinumtoxinA may provide additional therapeutic benefits in treating acute post-traumatic headaches, preventing their progression to persistent states and in reversing established persistent post-traumatic headaches.

## Supplementary Information

Supplementary Material 1.

## Data Availability

Supplementary material is available online. The datasets used and/or analyzed during the current study are available from the corresponding author on reasonable request.

## Abbreviations

CGRP: Calcitonin gene-related peptide
DCN: Descending control of nociception
mTBI: Mild traumatic brain injury
onabotA: OnabotulinumtoxinA
PGE2: Prostaglandin E2
PTH: Post-traumatic headache
