# Supplementary material for: OnabotulinumtoxinA inhibits dysregulation of descending pain modulation following mild traumatic brain injury in mice
Source: J Headache Pain. 2025 Oct 16;26(1):216. doi: 10.1186/s10194-025-02159-0 (PMC12532842; doi:10.1186/s10194-025-02159-0)
Supplement: Supplementary file 1 — Supplementary Material 1. [file 10194_2025_2159_MOESM1_ESM.docx]

**Supplementary material**

**Supplementary Table 1.** Summary of statistical analysis

| **Figures** | **Analysis** | **Test statistic** | **P-value** | **n** |
| --- | --- | --- | --- | --- |
| 1c | Two-way repeated-measures ANOVA, Tukey post-hoc test | F (21, 245) = 2.082^a^  F (7, 245) = 17.88^b^  F (3, 35) = 4.797^c^ | 0.0045^a^  <0.0001^b^  0.0067^c^ | Sham/vehicle = 14  Sham/onabotA = 7  mTBI/vehicle = 11  mTBI/onabotA = 7 |
| 1d | Two-way repeated-measures ANOVA, Tukey post-hoc test | F (21, 168) = 3.272^a^  F (7, 168) = 28.32^b^  F (3, 24) = 4.143^c^ | <0.0001^a^  <0.0001^b^  0.0168^c^ | Sham/vehicle = 9  Sham/onabotA = 9  mTBI/vehicle = 5  mTBI/onabotA = 5 |
| 1e | Two-way repeated-measures ANOVA, Tukey post-hoc test | F (21, 308) = 2.376^a^  F (7, 308) = 46.70^b^  F (3, 44) = 5.009^c^ | 0.0008^a^ <0.0001^b^  0.0045^c^ | Sham/vehicle = 10  Sham/onabotA = 14  mTBI/vehicle = 13  mTBI/onabotA = 11 |
| 1f | Two-way repeated-measures ANOVA, Tukey post-hoc test | F (21, 203) = 2.115^a^  F (7, 203) = 27.55^b^  F (3, 29) = 4.877^c^ | 0.0042^a^ <0.0001^b^  0.0072 ^c^ | Sham/vehicle = 11  Sham/onabotA = 6  mTBI/vehicle = 10  mTBI/onabotA = 6 |
| 1g | Two-way repeated-measures ANOVA, Tukey post-hoc test | F (21, 581) = 3.771^a^  F (7, 581) = 65.75^b^  F (3, 83) = 8.534^c^ | <0.0001^a^  <0.0001^b^  <0.0001^c^ | Sham/vehicle = 24  Sham/onabotA = 21  mTBI/vehicle = 24  mTBI/onabotA = 18 |
| 1h | Two-way repeated-measures ANOVA, Tukey post-hoc test | F (21, 399) = 4.217^a^  F (7, 399) = 53.04^b^  F (3, 57) = 7.795^c^ | <0.0001^a^  <0.0001^b^  0.0002^c^ | Sham/vehicle = 20  Sham/onabotA = 15  mTBI/vehicle = 15  mTBI/onabotA = 11 |
| 1i | One-way ANOVA, Tukey post-hoc test | F (3, 83) = 6.916 | 0.0003 | Sham/vehicle = 24  Sham/onabotA = 21  mTBI/vehicle = 24  mTBI/onabotA = 18 |
| 1j | One-way ANOVA, Tukey post-hoc test | F (3, 57) = 6.333 | 0.0009 | Sham/vehicle = 20  Sham/onabotA = 15  mTBI/vehicle = 15  mTBI/onabotA = 11 |
| 2b | Two-way repeated-measures ANOVA, Tukey post-hoc test | F (7, 112) = 0.3412^a^  F (7, 112) = 15.97^b^  F (1, 16) = 0.6117^c^ | 0.9333^a^  <0.0001^b^  0.4456^c^ | Sham = 8  mTBI = 10 |
| 2c | Two-way repeated-measures ANOVA, Tukey post-hoc test | F (7, 98) = 0.9028^a^  F (7, 98) = 7.987^b^  F (1, 14) = 0.2117^c^ | 0.5076^a^  <0.0001^b^  0.6525^c^ | Sham = 8  mTBI = 8 |
| 2d | Unpaired two-tailed Student t-test | t=0.1537, df=16 | 0.8798 | Sham = 8  mTBI = 10 |
| 2e | Unpaired two-tailed Student t-test | t=0.2359, df=14 | 0.8169 | Sham = 8  mTBI = 8 |
| 3b | Two-way repeated-measures ANOVA, Tukey post-hoc test | F (24, 560) = 1.417^a^  F (8, 560) = 34.12^b^  F (3, 70) = 2.900^c^ | 0.0910^a^  <0.0001^b^  0.0410^c^ | Sham/vehicle = 21  Sham/onabotA = 17  mTBI/vehicle = 20  mTBI/onabotA = 16 |
| 3c | Two-way repeated-measures ANOVA, Tukey post-hoc test | F (24, 240) = 4.056^a^  F (8, 240) = 50.02^b^  F (3, 30) = 3.880^c^ | <0.0001^a^ <0.0001^b^  0.0186^c^ | Sham/vehicle = 12  Sham/onabotA = 5  mTBI/vehicle = 10  mTBI/onabotA = 7 |
| 3d | One-way ANOVA, Tukey post-hoc test | F (3, 70) = 3.929 | 0.0119 | Sham/vehicle = 21  Sham/onabotA = 17  mTBI/vehicle = 20  mTBI/onabotA = 16 |
| 3e | One-way ANOVA, Tukey post-hoc test | F (3, 30) = 5.195 | 0.0052 | Sham/vehicle = 12  Sham/onabotA = 5  mTBI/vehicle = 10  mTBI/onabotA = 7 |
| 4b | Two-way repeated-measures ANOVA, Tukey post-hoc test | F (24, 304) = 2.412^a^  F (8, 304) = 46.43^b^  F (3, 38) = 5.751^c^ | 0.0003^a^  <0.0001^b^  0.0024^c^ | Sham/vehicle = 11  Sham/onabotA = 10  mTBI/vehicle = 15  mTBI/onabotA = 6 |
| 4c | Two-way repeated-measures ANOVA, Tukey post-hoc test | F (24, 304) = 3.616^a^  F (8, 304) = 79.47^b^  F (3, 38) = 4.720^c^ | <0.0001^a^  <0.0001^b^  0.0068^c^ | Sham/vehicle = 13  Sham/onabotA = 10  mTBI/vehicle = 7  mTBI/onabotA = 12 |
| 4d | One-way ANOVA, Tukey post-hoc test | F (3, 38) = 9.856 | <0.0001 | Sham/vehicle = 11  Sham/onabotA = 10  mTBI/vehicle = 15  mTBI/onabotA = 6 |
| 4e | One-way ANOVA, Tukey post-hoc tests | F (3, 38) = 9.725 | <0.0001 | Sham/vehicle = 13  Sham/onabotA = 10  mTBI/vehicle = 7  mTBI/onabotA = 12 |

Note: ^a^Interaction F- and P-values. ^b^Time F- and P-values. ^c^Treatment F- and P-values.
